# Redox Mediator: A New Strategy in Designing Cathode for Prompting Redox Process of Li–S Batteries

**DOI:** 10.1002/advs.201900958

**Published:** 2019-09-10

**Authors:** Xian Wu, Nannan Liu, Bin Guan, Yue Qiu, Maoxu Wang, Junhan Cheng, Da Tian, Lishuang Fan, Naiqing Zhang, Kening Sun

**Affiliations:** ^1^ State Key Laboratory of Urban Water Resource and Environment School of Chemistry and Chemical Engineering Harbin Institute of Technology Harbin 150001 China; ^2^ Academy of Fundamental and Interdisciplinary Sciences Harbin Institute of Technology Harbin 150001 China

**Keywords:** energy efficiency, kinetics, Li–S batteries, mediators, polysulfide

## Abstract

The multistep redox reactions of lithium–sulfur batteries involve undesirably complex transformation between sulfur and Li_2_S, and it is tough to spontaneously fragmentate polysulfides into shorter chains Li_2_S originating from the sluggish redox kinetics of soluble polysulfide intermediates, causing serious polarization and consumption of sulfur. In this work, 3,4,9,10‐perylenetetracarboxylic diimide (PTCDI)/G is employed as sulfur host to accelerate the conversion process between polysulfides and sulfur, which could facilitate the process of both charging and discharging. Moreover, PTCDI has strong adsorption capacity with polysulfides to restrain shuttle effect, resulting in promotional kinetics and cycle stability. A high initial capacity of 1496 mAh g^−1^ at 0.05 C and slight capacity decay of 0.009% per cycle at 5 C over 1500 cycles can be achieved. Moreover, the cathode could also achieve a high energy efficiency over 85% at 0.5 C. This research extends the knowledge into an original domain for designing high‐performance host materials.

## Graphical abstract

**A redox mediator is employed as sulfur host material for lithium–sulfur batteries**. The 3,4,9,10‐perylenetetracarboxylic diimide (PTCDI) acts as an electron–hole transfer agent to accelerate the redox of polysulfides into Li_2_S, and the polar PTCDI could restrain the shuttle effect of polysulfides. This strategy for designing cathodes enables high energy efficiency and good cycle stability of lithium–sulfur batteries.

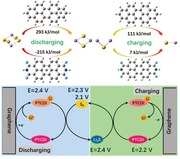

With the mounting requirement for energy density, the property of lithium‐ion batteries (LIB) has been steadily improved.^1^ However, as a determinate technology, the insertion‐compound electrode used in current LIB are getting to their ultimate charge storage capacities.^2^ Surpassing LIB, a high specific capacity of sulfur cathode combined with a lithium anode could provide a capacity of 1675 mAh g^−1^.^3^ This system has the ability to afford times higher energy density than the commercial LIB, which is promising to alternate current LIB as next generation energy storage device.

The redox processes of lithium–sulfur batteries involve complex multiple electrons conversion reaction, and the process inevitably generates soluble polysulfides intermediate, which would cause high charge transfer resistance and passivation of anode surface by migrated polysulfides.^4^ To address the problem, many reports were focused on designing cathode host materials,^5^ functional separators,^6^ and anode passivation layer to dispose the “shuttle issue,”^7^ which would perform an enhanced stability of batteries. Unfortunately, lithium–sulfur batteries also suffer sluggish kinetics caused by the high energy barrier of redox process,^8^, ^9^ and there is hardly effective way to satisfy the intrinsic stagnant redox process between S_8_ and Li_2_S. How to enhance the kinetics of the redox reaction of lithium–sulfur battery is still a critical challenge to promote the energy efficiency of batteries. Involving redox mediators (RMs) in electrolyte proposes an effective strategy to address the sluggish kinetic in lithium–oxygen (Li–O_2_) batteries and redox flow cell.^10^, ^11^, ^12^ This approach depends on electrochemical redox of RMs in solution, which can in turn chemically oxidize active materials on the surface of the electrodes. The additional charge transfer route beyond the localized interface enables homogeneous and complete oxidation of the electrode with a reduced overpotential. The redox potential is key factor in selecting RMs, and the study of employing RMs in Li–S batteries is still in its infancy.^13^

In this work, we employ 3,4,9,10‐perylenetetracarboxylic diimide (PTCDI) attached on the surface of reduced graphene oxide (PTCDI/G) as sulfur host to impelling polysulfide redox. The PTCDI could act as charge transfer agent to accelerate the redox of polysulfides into Li_2_S. As the potentials of the two redox couples of PTCDI just straddle the delithiation/lithiation potential of sulfur, PTCDI would suffice for both charging and discharging of Li–S batteries. Different from previous RMs additive, the insoluble PTCDI/G host could not only speed up the reaction by changing the reaction path of polysulfides and facilitate the process of both charge and discharge, but also stabilize polysulfides to restraining shuttle effect. To the best of our knowledge, this is the first report to introduce such redox mediator as sulfur host to come up with a new strategy for prompting redox process of lithium–sulfur reaction. The unique reaction mechanism could generate high energy efficiency of 85% at 0.5 C. Besides, amide bond in PTCDI could generate strong polar active site to immobilize polysulfides to mitigate the shuttle effect. The effectiveness on accelerating the transformation of polysulfides achieves a high initial specific capacity of 1496 mAh g^−1^ at 0.05 C and commendable low decay rate of 0.022% per cycle at 1 C.

PTCDI/G was fabricated from n‐type semiconductor PTCDI by solution phase self‐assembly method (**Figure** **1**). First, rGO solution was poured into a homogeneous PTCDI solution and kept stirring for 30 min, the PTCDI was precipitated at the interface of the two liquid phases (Figure S1, Supporting Information), and further induced self‐assembly by π–π interaction between PTCDI and rGO, which was proved by UV spectrum in Figure S2 in the Supporting Information. The maximum absorption (λ_max_) of PTCDI/G had a obvious red shift compared with bare PTCDI, this phenomenon could be explained by the fact that π–π stack would increase the energy of the π orbital and reduce the energy of the π* orbital, and then affect the absorption wavelength of the π–π* transition in the UV spectrum.^14^ The π–π interaction can likewise cause the shift of Raman spectrum. The Raman spectra of rGO had two characteristic peaks at 1360 and 1610 cm^−1^ (Figure S3, Supporting Information), representing the D band and G band of rGO,^15^ and the characteristic peaks of PTCDI were reflected at frequency regions of 1325, 1400, and 1600 cm^−1^.^16^ After compositing with rGO, the peaks of PTCDI all shifted to lower frequency, which similarly explains the existence of π–π interaction between PTCDI and rGO.^17^ Moreover, the color of bare PTCDI powder was puce, which turned into dark yellow after self‐assembly with rGO (Figure S4, Supporting Information).

**Figure 1:**
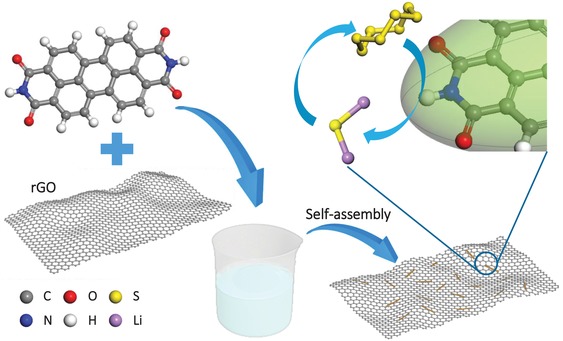
Schematic illustration of synthetic route for PTCDI/G composites.

The morphology of the PTCDI/G was revealed in **Figure** **2**a and Figure S5 in the Supporting Information. PTCDI nanoribbon was snapped on rGO surface, and the well interconnection between rGO and PTCDI ensure the unhindered electron transfer ability. X‐Ray Diffraction (XRD) in Figure 2b performed the crystal structure of PTCDI/G. Bare PTCDI possessed a typical crystal structure including (001), (201), and (202) lattice planes,^18^ and the structure remained unchanged after compositing with rGO and sulfur. Fourier transform infrared spectroscopy (FTIR) was displayed in Figure 2c to further analyze the PTCDI/G composites. In the IR spectra of PTCDI/G composite, the peaks appearing at around 1690 cm^−1^ could be related to the stretching modes of C=O in PTCDI,^19^ while the stretching modes of C=C in benzene correspond to four peaks between 1450 and 1600 cm^−1^, and the absorption around 1425 cm^−1^ were attributed by the C—N of amido bond. In contrast, the absorption of PTCDI/G over 3000 cm^−1^ corresponding to the stretching vibration of C—H. The results suggest that the PTCDI molecules are deeply composited with rGO network. The content of PTCDI in PTCDI/G was ≈50 wt% measured by thermogravimetric analysis (TGA) (Figure S6, Supporting Information), which was accordant to the proportion of raw materials. Moreover, the content of nitrogen was 3.6 wt% by XPS analysis, which also stated that the mass ratio of PTCDI was 50% in PTCDI/G.

**Figure 2:**
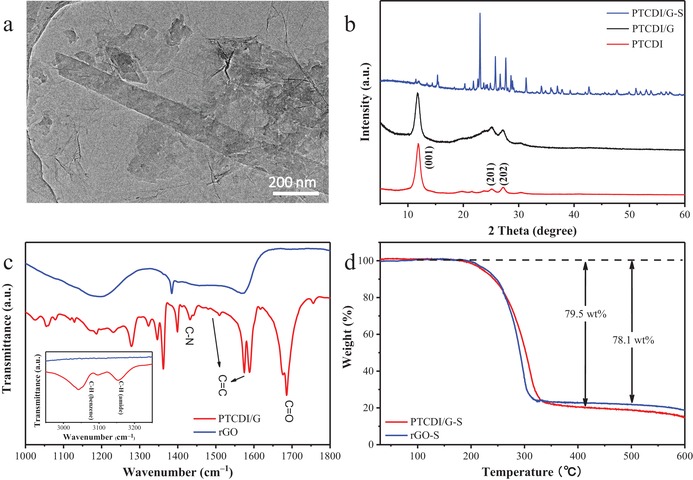
a) TEM image of PTCDI/G. b) XRD pattern of PTCDI, PTCDI/G. c) FT‐IR spectrum of PTCDI/G and rGO. d) TGA curves of PTCDI/G‐S and rGO‐S.

The chemical state of the PTCDI/G composite was further investigated by X‐ray photoelectron spectroscopy (XPS). As shown in Figure S7a in the Supporting Information, there was no additional heterogeneous elements in PTCDI/G composite. The C 1s XPS spectra can be decomposed into four peaks (Figure S7b, Supporting Information), representing the existence of C—C, C=O, C—N, COO^−^ in PTCDI and rGO. The high‐resolution N 1s XPS spectrum (Figure S2c, Supporting Information) revealed the presence of C—N and H—N in diimide group, and the peak of O 1s spectrum (Figure S7d, Supporting Information) was readily assigned to C=O, C—O, and OH^−^, ascribed to surface oxygenic functional groups of rGO, while partly C=O was attributable to diimide of PTCDI.

Cyclic voltammetric (CV) curve of bare PTCDI is exhibited in **Figure** **3** to manifest the redox process of PTCDI in Li–S system. There were two pairs of redox peaks for PTCDI during the voltage of 1.5–3 V (Figure 3a). The reduction of PTCDI occurred at 2.45 and 1.88 V, and the oxidation peaks appeared at 2.11 and 2.55 V with the following positive scanning. The peaks potential changed little and the CV curves were nearly identical with the cycle numbers increasing, indicating that bare PTCDI showed good cycle stability. Comparably, the CV curves of bare rGO had no redox peak within the voltage of 1.5–3 V (Figure S9, Supporting Information), illustrating that the unique redox reaction of PTCDI compared with carbon materials. The CV curves of PTCDI versus PTCDI/G‐S were performed in Figure 3b. The redox potentials of sulfur located exactly between the potentials of PTCDI, which was ideal for chemical reaction between PTCDI and sulfur.^12^ The schematically mechanism of PTCDI‐mediated Li–S redox reaction are described in Figure 3c. On discharge, PTCDI was reduced at the cathode of 2.45 V, and the generated reductive Li–PTCDI would transfer Li^+^ to polysulfides during the following discharge process, increasing the Li^+^ concentration around the polysulfides and accelerating reduction of polysulfides to form Li_2_S. On charge, Li–PTCDI oxidized at the cathode primarily, the PTCDI would accept the released Li^+^ from Li_2_S and react with Li_2_S to generate polysulfides and further convert back into S_8_ and regenerated Li–PTCDI.^10^, ^12^ The ability to detach Li^+^ of PTCDI could dynamically supply and receive Li^+^ for sulfur conversion during charging and discharging. To verify the hypothesis, theoretical studies were operated on G09 Gaussian package and aimed at understanding the reactivity between PTCDI and polysulfides. We calculated the Li_2_S_4_ decomposition process during discharge (Figure 3d). In general, the disintegration of Li_2_S_4_ into Li_2_S_2_ required absorbing thermodynamics Gibbs free energy (Δ*G*) of 293 kJ mol^−1^, which is observably less probable. When PTCDI was employed as sulfur host, PTCDI first reduced to Li–PTCDI at potential of 2.45 V, whereafter Li–PTCDI would transfer Li^+^ to Li_2_S_4_, producing PTCDI and Li_2_S_2_ and releasing as much as 215 kJ mol^−1^. The result suggested that the decomposition of Li_2_S_4_ would transform into thermodynamically spontaneous with the existing of Li–PTCDI. The influence of PTCDI on charging response was shown in Figure 3e. The solid–liquid phase conversion from Li_2_S to polysulfides was difficult to be spontaneous and demanded Δ*G* of 111 kJ mol^−1^. On the contrary, PTCDI would adopt Li^+^ of Li_2_S and expedite its disintegration with only an estimated Δ*G* of 7 kJ mol^−1^, which is much lower than that without PTCDI. The result stated that PTCDI could also facilitate the decomposition of Li_2_S during charging. The possible equations of the Li–S reaction process with PTCDI are shown in Figure 3f.

**Figure 3:**
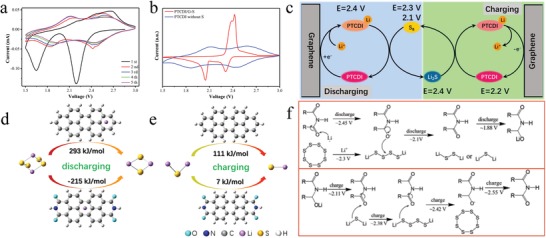
a) CV curves of PTCDI/G for the initial 5 cycles at a scan rate of 0.1 mV s^−1^, and b) corresponding CV comparison with PTCDI/G‐S. c) Schematic illustration of electrode reactions on discharge and charge with PTCDI mediator for Li–S batteries. Selected results of quantum chemistry calculations and reaction energies for reactions of d) Li_2_S_4_ decomposition and e) Li_2_S oxidation. f) The possible equations of the charge–discharge reaction process with PTCDI.

It is worth mentioning that although the bare PTCDI/G presented redox during cycles, it contributed negligible capacity (only about 15 mAh g^−1^ based on the mass of sulfur) for the battery (Figure S11b, Supporting Information), which only contributes ≈1% capacity compared with sulfur. Moreover, PTCDI/G had commendable rate performance, the capacity barely decayed from 0.1 to 5 A g^−1^ (Figure S12, Supporting Information), symbolizing that the PTCDI/G could maintain good charge transfer ability even at high rate test of Li–S batteries. To eliminate the influence of PTCDI dissolution in electrolyte, PTCDI/G‐S and Li foil were placed at the opposite sides of H‐type glass electrolytic cell (Figure S13a, Supporting Information), and the battery was cycled at 0.1 C for 10 cycles. The UV spectrum of electrolyte were performed in Figure S13b, there was no noticeable additional absorption peak before and after cycles. The phenomenon illustrates that the dissolution of PTCDI could be neglected during cycles.

The lithium polysulfides adsorption capability of PTCDI/G was performed by mixing 30 mg of PTCDI/G with 3 mL of 10 × 10^−3^ m Li_2_S_4_ solution.^20^ The color of the Li_2_S_4_ solution changed into colorless after aged for 15 min with PTCDI/G added, while the bare rGO group still remained dark brown. The concentration variation of Li_2_S_4_ solution was further characterized by UV−vis adsorption spectra (Figure S14, Supporting Information), and the obvious decrease of Li_2_S_4_ concentration testified the effective adsorption ability of PTCDI/G. To further characterize the interaction between polysulfides and PTCDI, the adsorbed PTCDI/G was analyzed by XPS in Figure S15 in the Supporting Information. Li 1s spectrum was displayed in Figure S15a in the Supporting Information, the formation of Li–N bond stated that amide of PTCDI had chemical adsorption ability with lithium polysulfides. In Figure S15b in the Supporting Information, the two pairs of S doublets accorded the terminal (S_T_^−1^) and bridge (S_B_^0^) atoms, which indicated that polysulfides still existed after adsorption, and the color fading of solution is not caused by the oxidation of polysulfides.^21^ While the peaks of sulfate at high energy were caused by the oxidation during sample preparation under air condition. The results illustrated that the PTCDI would provide chemical bonding to restrict the dissolution of polysulfides.

CV curves of symmetrical batteries were also tested to study the redox kinetics of polysulfides, and all the cathodes were prepared with similar areal density.^8^, ^22^ The control group without Li_2_S*_n_* testified that the capacitive current dedicated minor portion of the polarization profiles, which mainly came from redox current of polysulfides (Figure S16a, Supporting Information). After employing Li_2_S_4_, the battery with bare rGO cathode had an increased polarization current, while the PTCDI/G performed times higher electrochemical response current, proving that PTCDI–polysulfide interaction not only statically generated, but also dynamically accelerated the electrochemical reactions of Li–S batteries. The promotion of redox reaction was further investigated by a two‐electrode system with 4 × 10^−3^ m S_8_ dissolved into electrolyte to eliminate the interference from other confounding factors, such as sulfur loading, the thick of the cathode, and the infiltration of electrolyte (Figure S16b, Supporting Information).^23^ There were two pairs of redox peaks versus Li/Li^+^ for both rGO and PTCDI/G under such system. Compared with rGO, PTCDI/G electrode had a conspicuous enhancement of peak current, and the peak voltage of PTCDI neared equilibrium potential, proving that the redox reaction can easily proceed on the PTCDI/G surface. Circumstantially, rGO could promote the electron transfer of electrode, and PTCDI could adsorb polysulfides and accelerate the fragmentation of polysulfides. The accelerated polysulfide redox was also studied by the onset potential changes of three redox peaks, and the onset potentials were taken at a current density of 10 µA cm^−2^ beyond the baseline current, following a common definition employed in electrocatalysis (Figure S17, Supporting Information).^8^, ^24^ As shown in Figure S18a in the Supporting Information, PTCDI/G‐S had increased onset potentials of cathodic peaks and decreased that of the anodic peak compared with rGO‐S cathode, illustrating the promotional kinetics by the PTCDI mediator.

According to the above results, we were confident that PTCDI would construct a good electrochemical performance as sulfur host of Li–S batteries. The electrochemical properties of PTCDI/G‐S and rGO‐S were studied between the cut‐off voltages of 1.7–2.8 V. The sulfur content was 79.5 and 78.1 wt% in the composites of PTCDI/G‐S and rGO‐S, respectively, which was confirmed by TGA under N_2_ atmosphere (Figure 2d). Cyclic voltammetry (CV) curves were carried out to manifest the redox behaviors of the electrode materials (**Figure** **4**a), the peak at about 2.3 V assigned to the reduction of S_8_ to intermediate polysulfides (Li_2_S*_x_*, 4 ≤ *x* ≤ 8), while the peak at about 2.0 V was caused by the reduction of intermediate polysulfides to Li_2_S. The oxidation peak at around 2.4 V corresponded to the conversion of Li_2_S into S_8_. Compared with rGO‐S, PTCDI/G‐S would increase the potentials of cathodic peaks and concurrently decrease potential of the anodic peak (Figure S16b, Supporting Information), and the sharper redox peaks and lower overpotential for PTCDI/G‐S cathode gave the credit to the fast reaction kinetics during cycles. The comparison of rate performance is displayed in Figure 4b with the voltage range of 1.7–2.8 V. PTCDI/G‐S exhibiting a capacity of 1496, 1236, 1044, 895, 793, 605, and 461 mAh g^−1^ with a rate of 0.05, 0.1, 0.2, 0.5, 1.0, 2.0, and 5.0 C. When the current density turned back to 0.1 C, the discharge capacity of PTCDI/G‐S could recover to 1102 mAh g^−1^, manifesting a good structural stability even at high current rate. However, the discharge capacity of rGO‐S suffered a rapid decrease from 1242 to 235 mAh g^−1^ with the current density increase from 0.05 to 5 C. The result illustrated that the addition of PTCDI contributed higher capacity and lower polarization even at high current density. The charge–discharge voltage profiles of PTCDI/G‐S contained two distinct discharge plateaus and one charge plateau at high current density of 5 C (Figure S19, Supporting Information), manifesting that PTCDI/G‐S had an excellent rate performance. The voltage plateaus of PTCDI/G‐S had negligible change with the increment of current density compared with rGO‐S (Figure S20, Supporting Information), which were contributed by the accelerating of charge transfer on account of PTCDI molecular existence.

**Figure 4:**
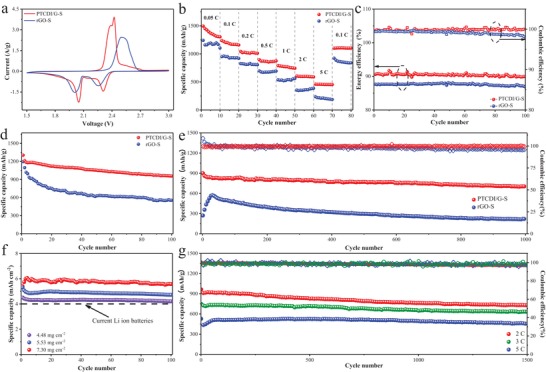
Electrochemical performance of Li–S batteries with PTCDI/G‐S cathode. a) CV curves and b) rate capability of Li–S batteries. c) Energy efficiency and Coulombic efficiency at 0.5 C, and d) cycling performance at 0.2 C. e) Cycling performance and Coulombic efficiency at 1 C. f) Cycling performance at 0.2 C with different sulfur loading cathodes. g) Long‐term cycling performances at 2, 3, and 5 C with PTCDI/G‐S cathodes.

Figure S21 in the Supporting Information revealed the galvanostatic charge–discharge behavior of PTCDI/G‐S cathode at 0.2 C. In accordance with the typical CV curves, the curves of both PTCDI/G‐S and rGO‐S incorporated with two plateaus during the discharge process and one plateau for the charging process. The difference was that PTCDI/G‐S cathode had a higher initial discharge capacity and smaller overpotential than rGO‐S. The PTCDI/G‐S displayed an initial discharge capacity of 1305 mAh g^−1^, and maintained 958 mAh g^−1^ after 100 cycles (Figure 4d). However, rGO‐S cathode suffered a rapid decrease from 1198 to 556 mAh g^−1^ after 100 cycles at 0.2 C. Energy efficiency, defined as the specific value between discharge specific energy and charge specific energy, is a more crucial factor for practical large‐scale lithium–sulfur batteries compared with Coulombic efficiency. Energy efficiency does not only represented the capacity ratio of discharging/charging, but also took account of plateau polarization of potential, and the higher energy efficiency would acquire less energy loss during electrochemical conversion process. As exhibited in Figure 4c, PTCDI/G‐S had a similar but slight higher Coulombic efficiency (over 99%) compared with rGO‐S at 0.5 C. However, the alleviative overpotential of PTCDI/G‐S resulted in promoted energy efficiency from 87% up to nearly 91% (Figure S22a, Supporting Information). The voltage gap at various current density is shown in Figure S22b in the Supporting Information, where PTCDI/G‐S had an assuasive overpotential especially at high current density. The promoted energy efficiency was significant for high‐efficiency Li−S batteries. Furthermore, PTCDI/G‐S performed stable long‐term cycle performance with discharge capacity variation from 900 to 700 mAh g^−1^ after 1000 cycles at a high current density of 1 C, and the capacity‐fading rate was as low as 0.022% per cycle (Figure 4e), while the rGO‐S cathode suffered a rapid capacity decay rate of 0.062%. The result stated that the unique structure of PTCDI/G possessed good electrical conductivity and strong chemical adsorption ability, resulting in low fading rate and high Coulombic efficiency.

High area capacity is another factor for further practical application; Figure 4f illustrated the cycle performance with different suflur loading at 0.2 C. The battery contributed a high initial capacity of 4.5 mAh cm^−2^ with sulfur loading of 4.48 mg cm^−2^, and the discharge capacity could reached 5.6 mAh cm^−2^ after 100 cycles with sulfur loading of 7.3 mg cm^−2^, which was much higher than current lithium ion batteries. The long‐term cycle stability of PTCDI/G‐S cathode at various rates were demonstrated in Figure 4g, the batteries could maintain a high specific capacity even at high rate of 5 C after 1500 cycles, indicative of classy cycle stability and rate performance. The electrolyte/electrode ratio (E/S) was also a significance ingredient for closer practical application. As shown in Figure S23 in the Supporting Information, the battery could retain two obvious plateaus under lean electrolyte condition of E/S = 6. The PTCDI/G‐S cathode provided high initial capacity of 1086 mAh g^−1^, and kept 870 mAh g^−1^ after 100 cycles. Compared with the other reports (Table S1, Supporting Information), our PTCDI/G host delivered better rate performance and cycle stability.

The electrochemical performance of the electrodes was further characterized by electrochemical impedance spectroscopy (EIS). All Nyquist plots of both cathodes contained a semicircle at high‐to‐medium frequency region and an inclined line at low frequency (Figure S24a, Supporting Information). The semicircle corresponded to charge transfer resistance (*R*_ct_) and the oblique line responded to the Warburg‐like impedance (W). The smaller *R*_ct_ of PTCDI/G‐S reflected the prosperous reactivity and kinetics at the open circuit potential. It is noteworthy that the *R*_ct_ values of PTCDI/G was diminished with the cycles proceed (Figure S24b, Supporting Information), which was mainly due to the rearrangement of sulfur in the cathode to shorter the pathway between the sulfur and host materials. On the contrary, the rGO‐S appeared additional semicircle after cycles; the emerging semicircle was associated with the Li_2_S deposition on the surface of anode caused by the dissolution of polysulfides.

The galvanostatic intermittent titration technique (GITT) was utilized to further study the changes in internal resistance of PTCDI/G‐S and rGO‐S electrodes. A constant current density of 0.02 C was applied for 60 min to obtain the closed‐circuit voltage (CCV) and then turned off for 60 min to measure the quasi‐open circuit voltage (QOCV). Figure S25a in the Supporting Information demonstrated the discharged transient voltage profiles, the changes in potentials in the wake of removing the current remained small in the high‐voltage region, whereas the CCV and QOCV had apparent separation with subtracting the current in low‐voltage region especially for rGO‐S, illustrating the polarization phenomenon is more obvious in this region. It is worth mentioning that the higher discharge plateau and slighter change in voltages after removing the current of PTCDI/G‐S was caused by its faster charge transfer, and the lower polarization compared with rGO‐S at the lower voltages gave the credit to the faster conversion dynamics.^25^ The internal resistances of the batteries during discharged were evolved from the difference between CCV and QOCV in each voltage transient.^26^ As shown in Figure S25b in the Supporting Information, the internal resistances of the first plateau showed a negligible change during dissolution of S into polysulfides; however, in the second plateau region, internal resistances increased suddenly by the deposition of insulated Li_2_S at cathode, and resistances kept increasing due to the loss of electron transfer active sites during the subsequent discharge. The lower resistances of PTCDI/G‐S illustrate that the distribution of produced Li_2_S is more uniform within the PTCDI/G‐S cathode.

Significantly, the electrochemical performance of the battery was easily optimized by altering the radio of PTCDI and rGO. The different PTCDI content composites were named as PG21, PG11, and PG12 based on the initial PTCDI and rGO radio. As performed in Figure S26 in the Supporting Information, PG12 revealed a higher initial discharge capacity of 1225 mAh g^−1^, but a poor cycle ability; however, PG21 performed a rare changed capacity after cycles but low initial discharge capacity of 1003 mAh g^−1^. The phenomenon stated that the higher PTCDI content was benefit for cycle stability. The large specific area of rGO was beneficial for uniform distribution of sulfur, and the better electron transfer ability could aggrandize the initial capacity of the batteries.

In conclusion, we design and synthesize PTCDI/G as an ideal sulfur host material for lithium–sulfur battery, which not only dynamically enhances polysulfide redox reactions by mediating charge transfer, but also provides strong adsorption capacity to polysulfides. The employment of such mediator offers prominent advantages on regulating the path of charge transfer, remitting heterogeneous polysulfides deposition, and accelerating the reaction kinetics of the electrochemical reaction during cycles. Meanwhile, the rGO with high specific area is beneficial for uniform distribution of sulfur and electron transfer. Based on the above advantages, PTCDI/G composite produces magical effects on sulfur cathodes, delivering a superior energy efficiency (85% at 0.5 C), highlighted specific capacity (1496 mAh g^−1^ at 0.05 C), and considerable cycle stability (capacity decay of 0.022% per cycle at 1 C). The concise and effective sulfur host cathode extends the redox chemistry of sulfur cathodes and realizes a promotable method to enhance the overall reaction process of Li–S batteries. Consequently, this new electrode constructive strategy inaugurates a novel design concept and inspires the development of high‐performance lithium–sulfur batteries.

## Conflict of Interest

The authors declare no conflict of interest.

## Supporting information

Supplementary
