# Supplementary material for: Redox Mediator: A New Strategy in Designing Cathode for Prompting Redox Process of Li–S Batteries
Source: Adv Sci (Weinh). 2019 Sep 10;6(21):1900958. doi: 10.1002/advs.201900958 (PMC6839637; doi:10.1002/advs.201900958)
Supplement: Supplementary file 1 — Supplementary [file ADVS-6-1900958-s001.pdf]

Copyright WILEY-VCH Verlag GmbH & Co. KGaA, 69469 Weinheim, Germany, 2019.

ADVANCED SCIENCE Open Access

Supporting Information

for *Adv. Sci.,* DOI: 10.1002/advs.201900958

Redox Mediator: A New Strategy in Designing Cathode for Prompting Redox Process of Li–S Batteries

Xian Wu, Nannan Liu, Bin Guan, Yue Qiu, Maoxu Wang, Junhan Cheng, Da Tian, Lishuang Fan, Naiqing Zhang,* and Kening Sun**

Copyright WILEY-VCH Verlag GmbH & Co. KGaA, 69469 Weinheim, Germany, 2019.

Supporting Information

Title Redox mediator: a new strategy in designing cathode for prompting redox process of Li-S batteries

Xian Wu, Nannan Liu, Bin Guan, Yue Qiu, Da Tian, Maoxu Wang, Junhan Cheng, Lishuang Fan, Naiqing Zhang* and Kening Sun**

Experimental Section

Synthesis of rGO: The GO was prepared by modified Hummers method, and the ascorbic acid was utilized as reduced agent. Detailedly, 25mg ascorbic acid was add into 70mL 1mg/mL GO at 95 °C and continuous stirred for 1h to obtain reduced graphene oxides (rGO).

Preparation of PTCDI/G: The homogeneous solution of PTCDI was prepared by ultrasonic it in sulfuric acid (10 mg/mL) for 30 min and subsequently a certain amount of rGO solution was added into the PTCDI solution to precipitate PTCDI at the interface of two phase, and further induced the self-assembly by π-π interaction of PTCDI with rGO. The complete formation of the PTCDI anchored on rGO were observed within 30 min. The dark brown powder was collected by centrifuging and freeze drying for 1 day.

Synthesis of Sulfur Composites: In order to synthesize PTCDI/G-S, the sulfur was composited with PTCDI/G with a simple melt-diffusion method. Briefly, 80 mg sulfur was dissolved into a certain amount of CS_2, and 30 mg PTCDI/G was added into above solution with continuous stirring at 60 °C. The obtained powder was then transferred into a sealed vessel and heated at 155 °C for 12 h. The rGO-S was prepared with the same method but using rGO rather than PTCDI/G.

Characterization of the materials: TEM (G2 F20FEI Tecnai G2 F20 microscope at 200 kV) conducted the morphology of the materials. The XRD pattern was applied to analyze the crystal structure of the materials by PANalytical X'Pert PRO, monochromated Cu K_α

radiation 40 mA, 40 kV. XPS was employed to analysis the compositions and chemical states with a PHI-5000 VersaProbe X-ray photoelectron spectrometer using an Al Kα X-ray radiation. The specific surface area, pore volume and N_2 adsorption/desorption isotherms were measured by an ASAP 2020 (Micromeritics).

Computation method: All calculations were performed using the Gaussian 09 software package.[1] The geometry optimizations were accomplished without symmetry constraints using DFT method and 6-31G** basis set for all atoms.

Visual test: A 10 mmol/L Li_2S_4 solution was prepared by adding sulfur and Li_2S at a molar ratio of 1:3 in 1,2-dimethoxymethane/1,3-dioxolane (DME/DOL) (1:1 by volume) solvent followed by vigorous magnetic stirring at room temperature till the solid dissolve into a uniform solution. 30 mg of PTCDI/G and rGO powders were added into 3 mL solution respectively and aged for times to observe the color of the solution.

Electrochemical tests: The cathodes were prepared with a mass radio of 8:1:1 of sulfur composite, Super P, and PVDF. The obtained surly was coated onto the Al foil and dried at 60°C for 12 h, and the areal density of sulfur was about 1 mg/cm^2. The coin batteries were assembled in an glovebox with argon-filled, and the electrolyte was constituted of 1.0 M bis(trifluoro-methane)sulfonamide lithium (LiTFSI) in solvent of 1,2-dimethoxyethane (DME) and 1,3-dioxolane (DOL) (1:1 v/v, with 2.0 wt % $LiNO_3$). The amount of electrolyte addition was 6-10 μL/mg based on the mass of the sulfur in the cathode. The galvanostatic charge–discharge process was measured on Neware with a potential window of 1.7−2.8 V. The cyclic voltammetry and AC impedance were measured on a CHI-660 electrochemical workstation.

Supplementary Figures and Tables

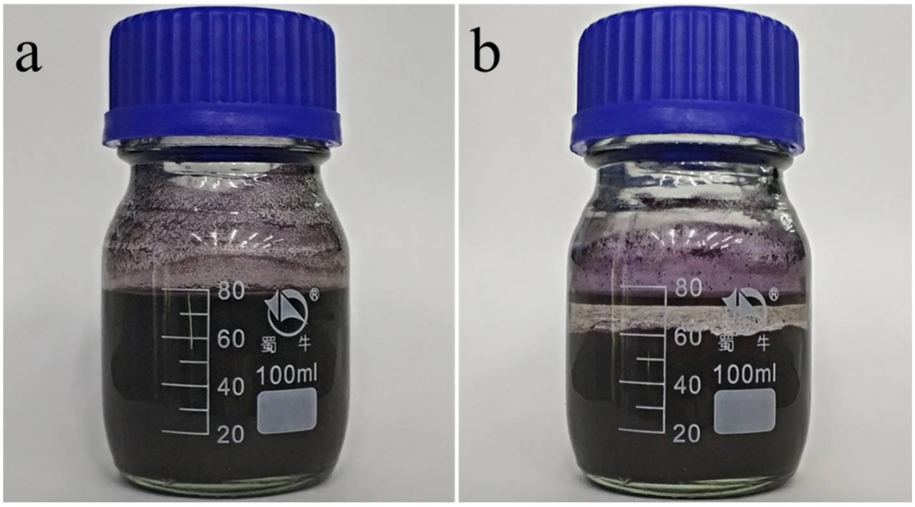

Figure S1. Optical photograph of (a) mixed and (b) precipitated PTCDI/GO solution.

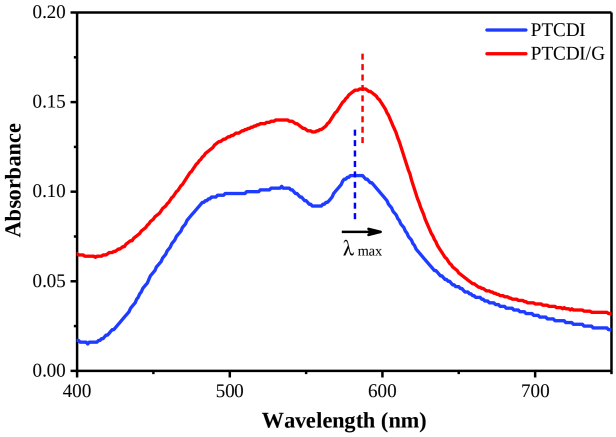

Figure S2. UV-vis spectrum of PTCDI and PTCDI/G in ethanol solution.

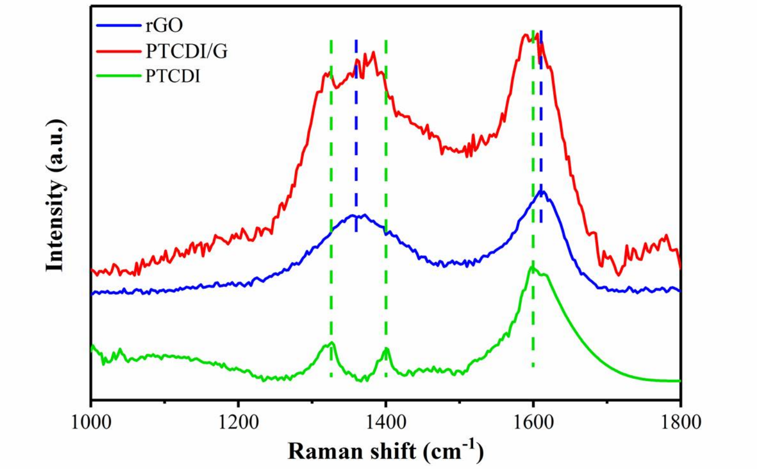

Figure S3. Raman spectra of PTCDI, rGO, and PTCDI/G.

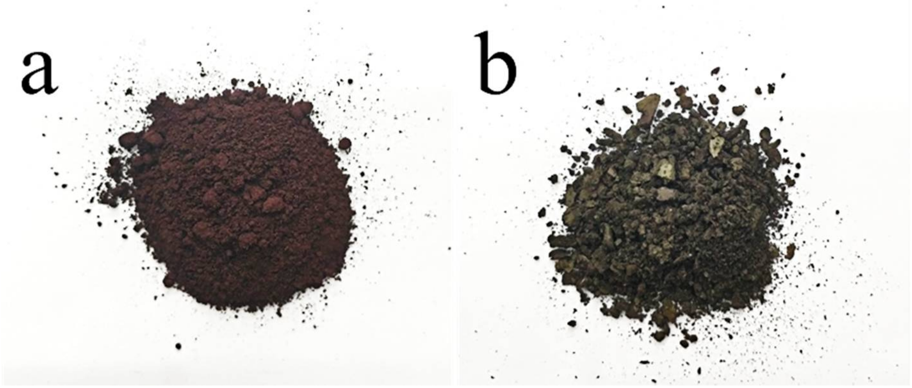

Figure S4. Optical photographs of (a) commercial PTCDI powder and (b) PTCDI/G powder.

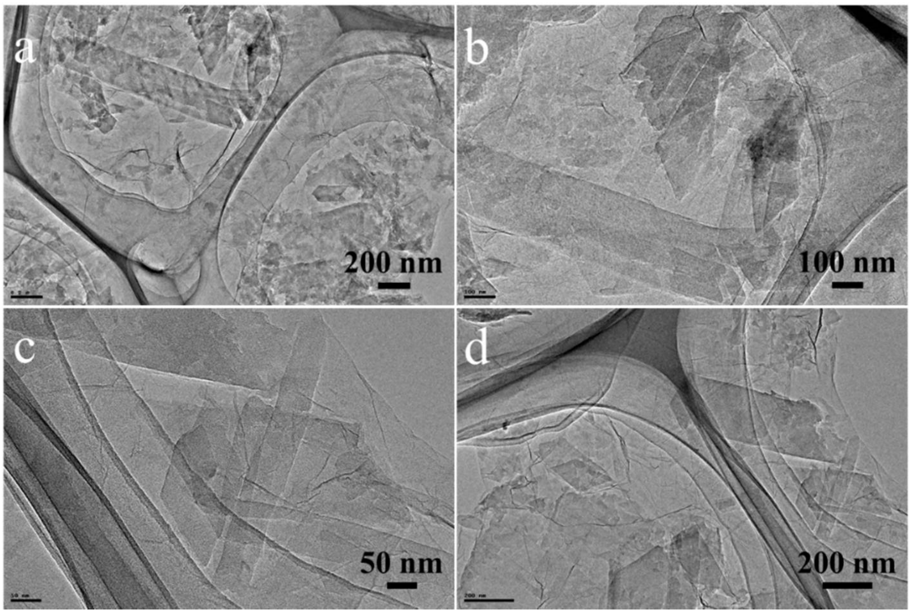

Figure S5. TEM images of PTCDI/G with different magnification.

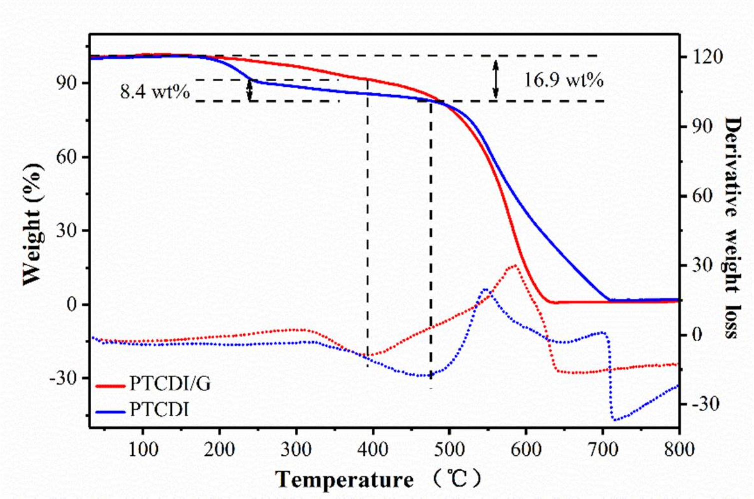

Figure S6. TG-DTA curves of PTCDI and PTCDI/G.

The TGA curve of bare PTCDI had two obvious mass loss stages corresponding to the preliminary carbonization and further oxidation process. The first stage was ended at 474 °C,

and the mass decrease about 16.9 wt%. While the first step of weight loss was ascribed by the evaporation of water in rGO at 200 °C, and carbonation process only offered 8.4 wt% weight loss for PTCDI/G, representing that the content of PTCDI in PTCDI/G was approximately 50 wt%, which was accordant to the proportion of raw materials.

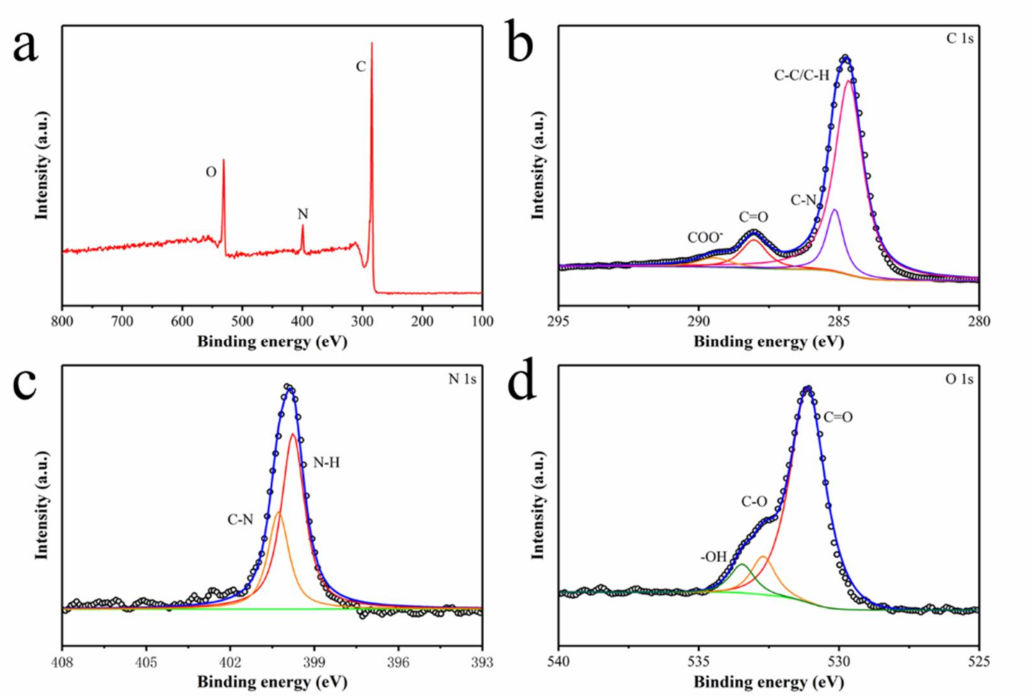

Figure S7. XPS spectra of PTCDI/G: (a) full survey spectra; (b) C 1s; (c) N 1s, and (d) O 1s.

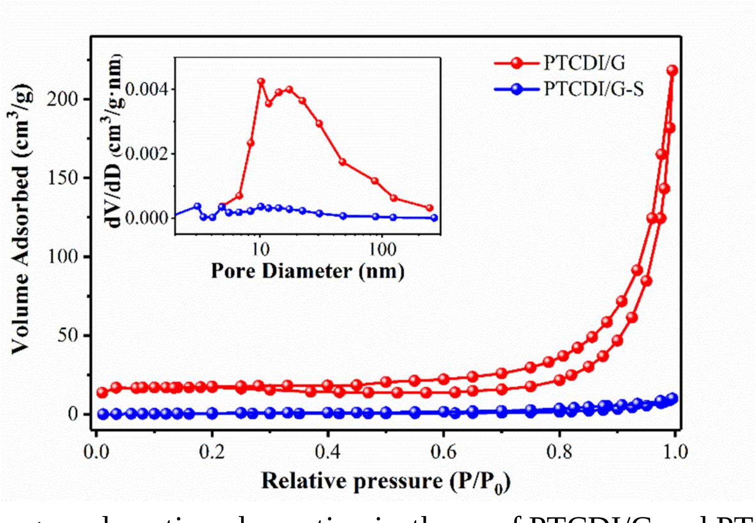

Figure S8. Nitrogen adsorption–desorption isotherm of PTCDI/G and PTCDI/G-S. Inset image shows the pore size distribution.

The N_2 adsorption/desorption isotherm showed that the specific surface area of PTCDI/G was 46.3 m^2/g, which was much higher than 1.3 m^2/g of PTCDI/G-S, and the pore volume of PTCDI/G reduced about 95 % after sulfur infusion. The results indicated that sulfur totally penetrated into the pores of PTCDI/G.

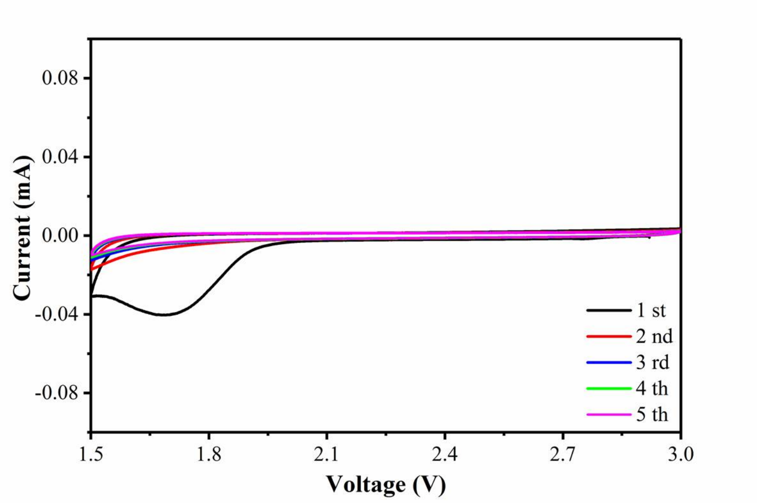

Figure S9. CV curves of bare rGO with a scanning rate of 0.1 mV/s.

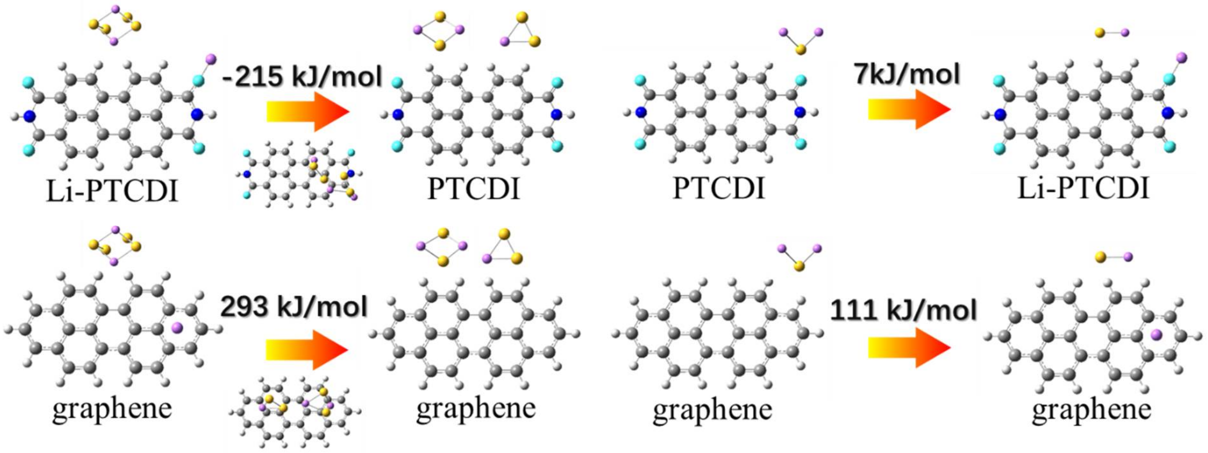

Figure S10. Geometric models for reactants and products of selected results of quantum chemistry calculations.

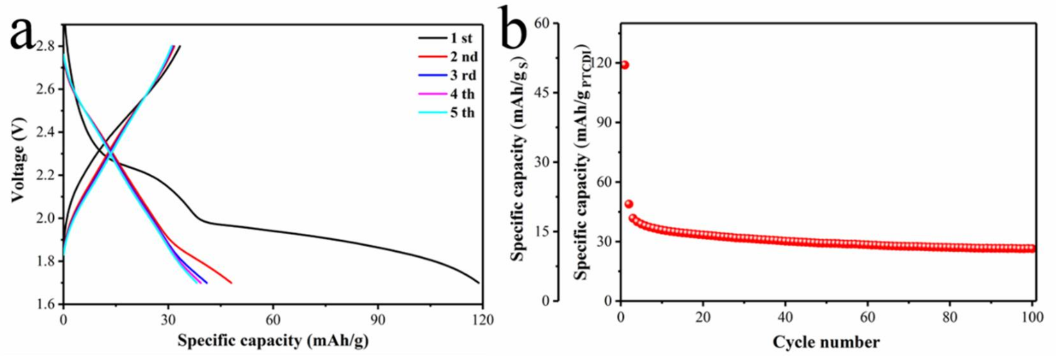

Figure S11. (a) Galvanostatic charge-discharge curves and (b) discharge capacity of bare PTCDI/G.

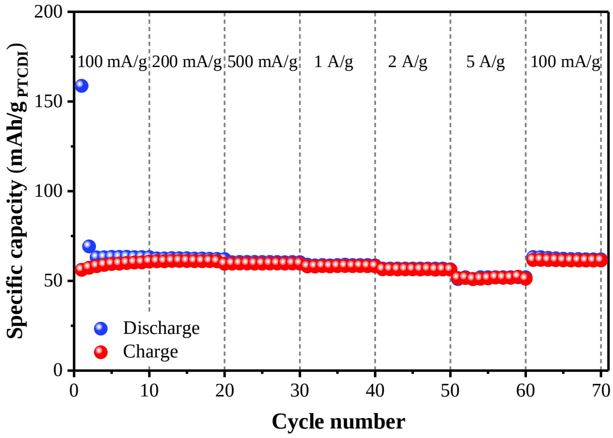

Figure S12. Rate performacne of PTCDI/G.

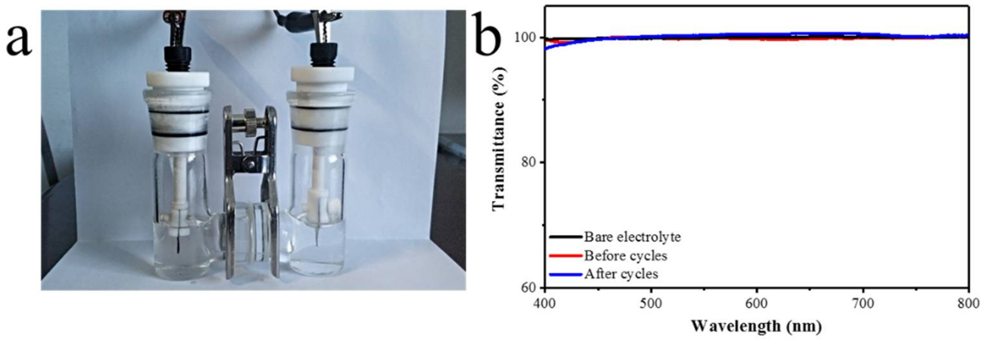

Figure S13. PTCDI dissolution test: (a) Optical photograph and (b) UV-vis spectrum of electrolyte.

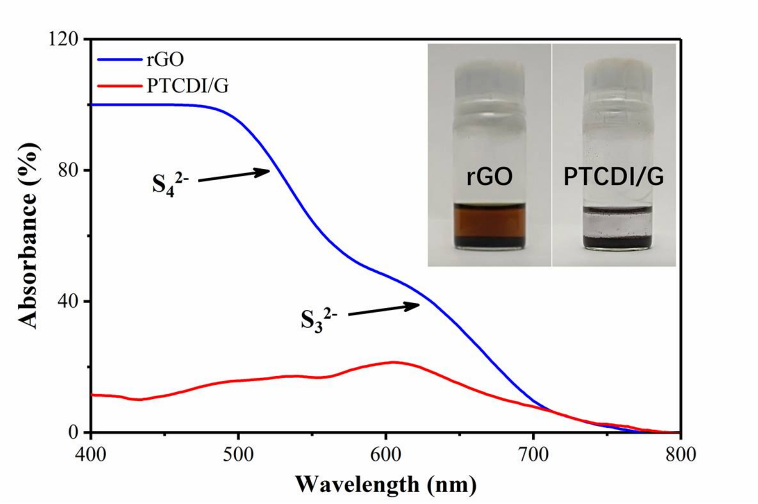

Figure S14. UV-vis absorption spectrum of Li_2S_4 solution after addition of rGO and PTCDI/G. Inset image shows a photograph of a Li_2S_4 solution after addition of rGO-S and PTCDI/G.

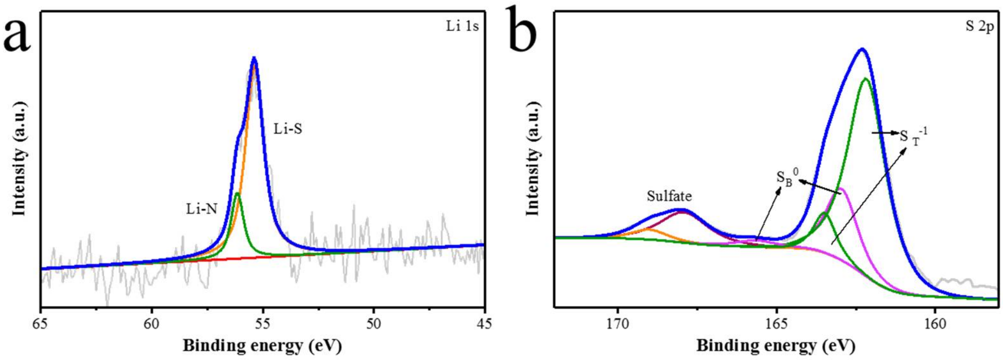

Figure S15. XPS spectra of PTCDI/G after adsorbed Li_2S_4: (a) Li 1s and (b) S 2p.

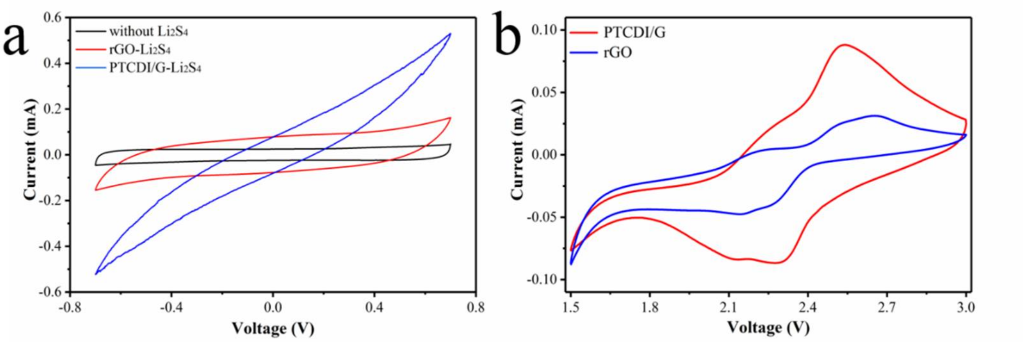

Figure S16. CV curves of (a) symmetrical batteries, and (b) sulfur redox reaction on PTCDI/G and rGO.

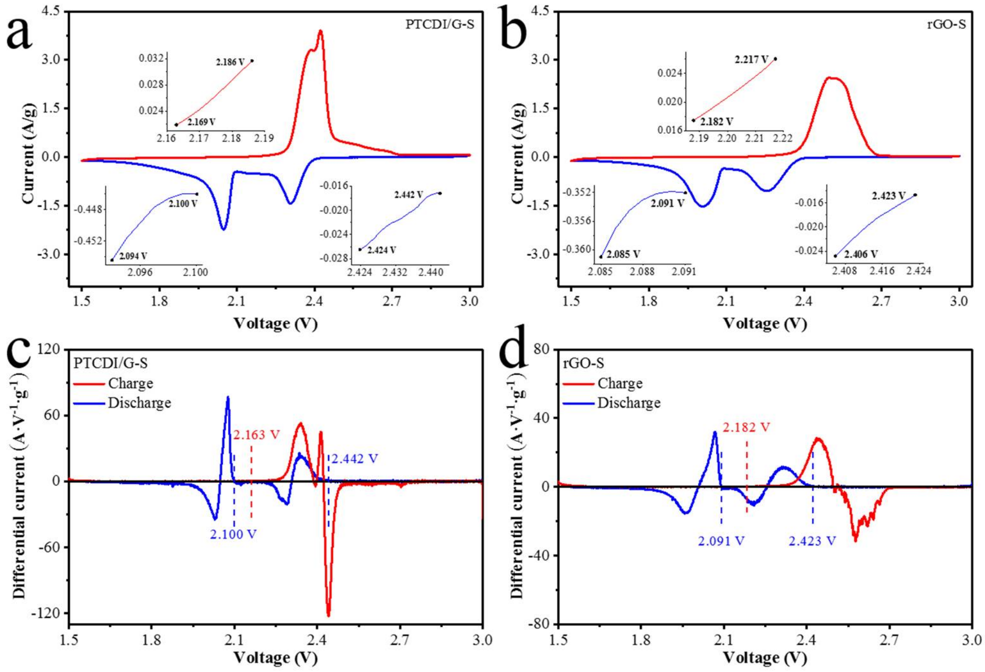

Figure S17. (a–c) CV curves and (d–f) differential current curves of (a, c) PTCDI/G-S, (b, d) rGO-S cathodes (Inset: corresponding onset potentials of redox peaks). The baseline current densities of the batteries are defined as the values before the redox peaks, where the values of differential current are infinitesimal.

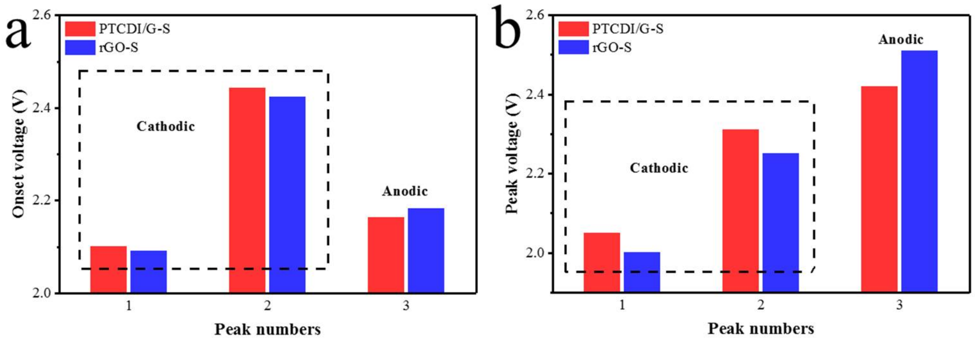

Figure S18. (a) Onset voltages and (b) plateau voltages of CV curves for rGO-S and PTCDI/G-S.

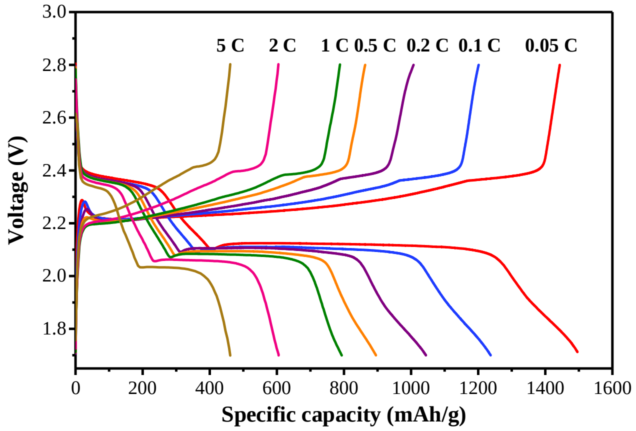

Figure S19. The charging and discharging curves of PTCDI/G-S at various rates.

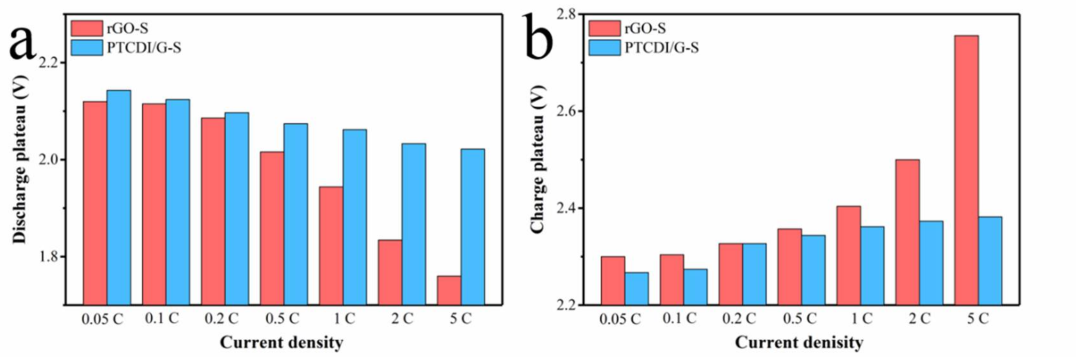

Figure S20. (a) Discharge plateau voltages and (b) charge plateau voltages of rGO-S and PTCDI/G-S.

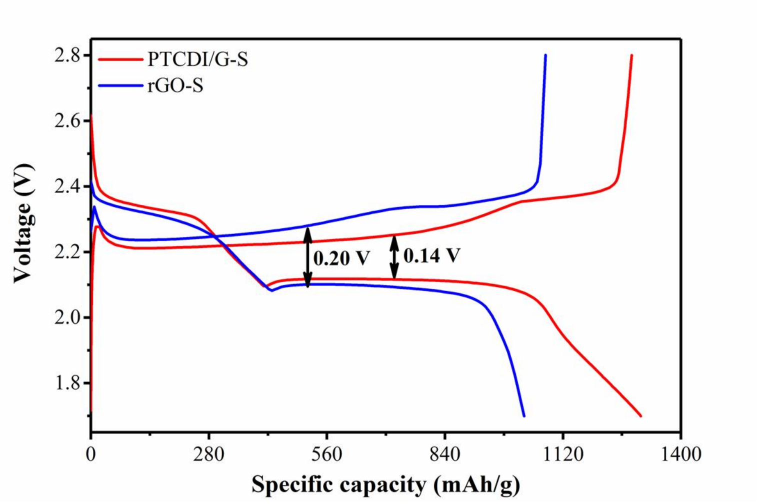

Figure S21. Charge/discharge curves at 0.2 C for rGO-S and PTCDI/G-S.

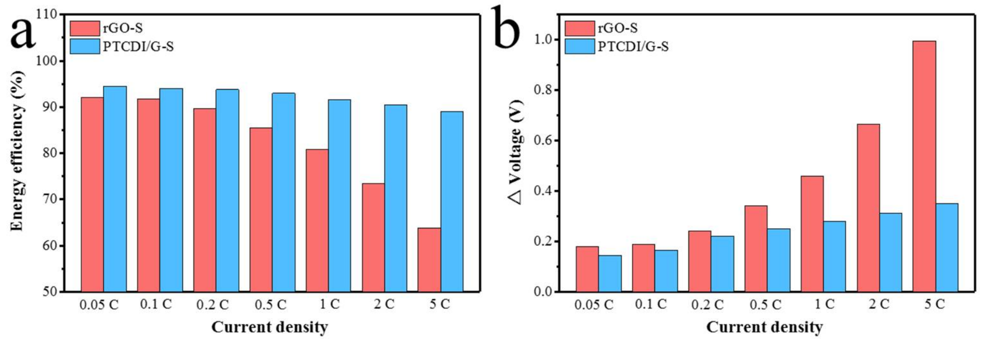

Figure S22. (a) Energy efficiency and (b) overpotential of rGO-S and PTCDI/G-S at different current densities.

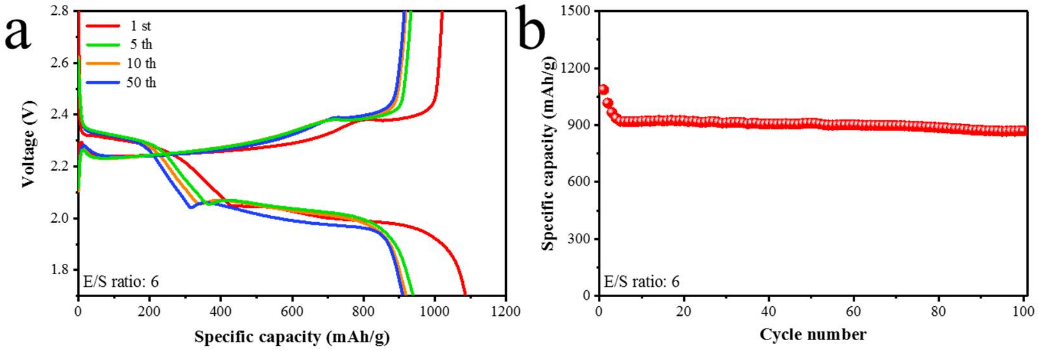

Figure S23. (a) Charge/discharge curves and (b) cycle performance at 0.2 C under electrolyte/electrode ratio of 6.

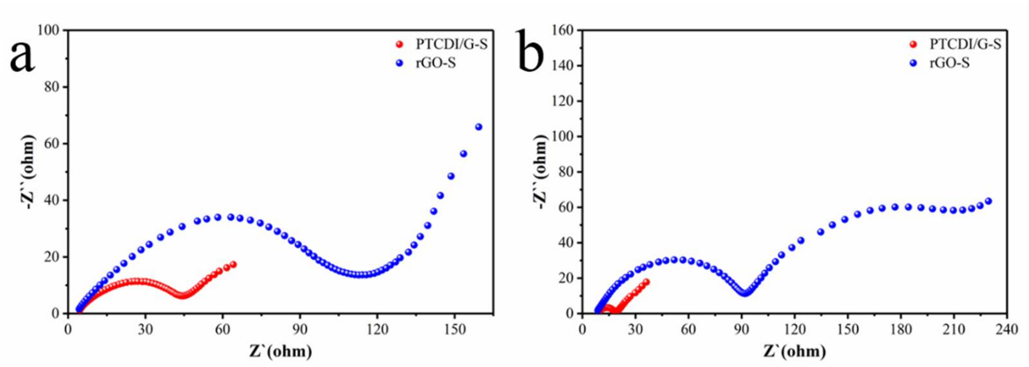

Figure S24. EIS spectra of rGO-S and PTCDI/G-S (a) before and (b) after cycles.

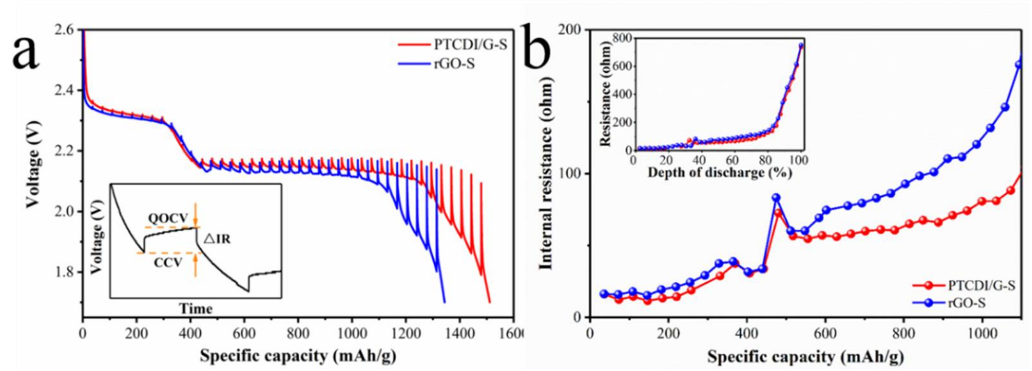

Figure S25. (a) Transient voltage profiles obtained with GITT (Inset: A typical voltage transient traced in one current pulse cycle). (b) The evolution of internal resistance calculated from the GITT.

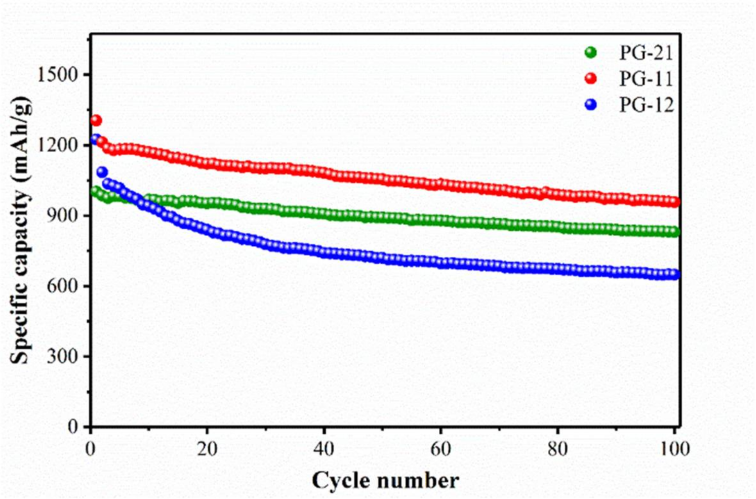

Figure S26. Cycling performance of PTCDI/G-S with different rGO contents.

Table S1. Electrochemical performance of Li-S batteries based on different host materials.

Sample	Sulfur content	Rate (C)	Cycle number	Capacity remained	Decay per cycle	Ref.
rGO film	56	1	500	681	0.69	[2]

Porous Graphene	68	0.5	150	879	0.11	[3]
Porous Carbon Nanosheets	74	1	100	866	0.25	[4]
N-Hollow Carbon Spheres	65	0.5	200	520	0.19	[5]
N,B-Graphene	70	1	250	588	0.08	[6]
N,S-Carbon	70	2	1100	365	0.052	[7]
Oxygenated Carbon Nitride	56	0.5	500	323	0.09	[8]
Amino-graphene	69	0.5	350	650	0.07	[9]
PTCDI/G	80	1	1000	700	0.022	This work

Reference

[1] Frisch GWT MJ, Schlegel HB, Scuseria GE, Robb MA, Cheeseman JR and Scalmani G and Barone V and Mennucci B and Petersson GA and Nakatsuji H and Caricato M and Li X and Hratchian HP and Izmaylov AF and Bloino J and Zheng G and Sonnenberg JL and Hada M and Ehara M and Toyota K and Fukuda R and Hasegawa J and Ishida M and Nakajima T and Honda Y and Kitao O and Nakai H and Vreven T and Montgomery, {Jr.}., JA and Peralta JE and Ogliaro F and Bearpark M and Heyd JJ and Brothers E and Kudin KN and Staroverov VN and Kobayashi R and Normand J and Raghavachari K and Rendell A and Burant JC and Iyengar SS and Tomasi J and Cossi M and Rega N and Millam JM and Klene M and Knox JE and Cross JB and Bakken V and Adamo C and Jaramillo J and Gomperts R and Stratmann RE and Yazyev O and Austin AJ and Cammi R and Pomelli C and Ochterski JW and Martin RL and Morokuma K and Zakrzewski VG and Voth GA and Salvador P and Dannenberg JJ and Dapprich S and Daniels AD and Farkas Ö and Foresman JB and Ortiz JV and Cioslowski J and Fox DJ. Gaussian~09 {R}evision {D}.01.

[2] J. Cao, C. Chen, Q. Zhao, N. Zhang, Q. Lu, X. Wang, Z. Niu, J. Chen, *Adv. Mater.* **2016**,

28, 9629.

[3] C. Tang, B.-Q. Li, Q. Zhang, L. Zhu, H.-F. Wang, J.-L. Shi, F. Wei, *Adv. Funct. Mater.* **2016**, *26*, 577.

[4] X. a. Chen, Z. Xiao, X. Ning, Z. Liu, Z. Yang, C. Zou, S. Wang, X. Chen, Y. Chen, S. Huang, *Adv. Energy Mater.* **2014**, *4*, 1301988.

[5] G. Zhou, Y. Zhao, A. Manthiram, *Adv. Energy Mater.* **2015**, *5*, 1402263.

[6] S. Yuan, J. L. Bao, L. Wang, Y. Xia, D. G. Truhlar, Y. Wang, *Adv. Energy Mater.* **2016**, *6*, 1501733.

[7] Q. Pang, J. Tang, H. Huang, X. Liang, C. Hart, K. C. Tam, L. F. Nazar, *Adv. Mater.* **2015**, *27*, 6021.

[8] J. Liu, W. Li, L. Duan, X. Li, L. Ji, Z. Geng, K. Huang, L. Lu, L. Zhou, Z. Liu, W. Chen, L. Liu, S. Feng, Y. Zhang, *Nano lett.* **2015**, *15*, 5137.

[9] Z. Wang, Y. Dong, H. Li, Z. Zhao, H. B. Wu, C. Hao, S. Liu, J. Qiu, X. W. Lou, *Nat. Commun.* **2014**, *5*, 5002.
